# Association of lung function with cardiovascular risk: a cohort study

**DOI:** 10.1186/s12931-018-0920-y

**Published:** 2018-11-06

**Authors:** Bin Wang, Yun Zhou, Lili Xiao, Yanjun Guo, Jixuan Ma, Min Zhou, Tingming Shi, Aijun Tan, Jing Yuan, Weihong Chen

**Affiliations:** 1 Department of Occupational & Environmental Health, School of Public Health, Tongji Medical College, Huazhong University of Science and Technology, Wuhan, 430030 Hubei China; 2 Key Laboratory of Environment and Health, Ministry of Education & Ministry of Environmental Protection, and State Key Laboratory of Environmental Health (Incubating), School of Public Health, Tongji Medical College, Huazhong University of Science and Technology, Wuhan, 430030 Hubei China; 3 Hubei Provincial Key Laboratory for Applied Toxicology, Hubei Provincial Center for Disease Control and Prevention, Wuhan, 430079 Hubei China; 4 Zhuhai Center for Disease Control and Prevention, Zhuhai, 519060 Guangdong China

**Keywords:** Lung function, Chronic obstructive pulmonary disease, Cardiovascular disease, Cohort study

## Abstract

**Background:**

The potential effects of pulmonary dysfunction on cardiovascular diseases (CVD) are receiving attention. We aimed to investigate and quantify the cross-sectional and longitudinal associations between lung function and overall cardiovascular risk among Chinese general population.

**Methods:**

We studied 4019 participants from the Wuhan-Zhuhai cohort, with a follow-up of 3 years. A multivariable risk algorithm generated from the Framingham study was used to calculate individuals’ overall cardiovascular risk i.e. 10-Year CVD Risk, which was further classified into 2 categories: low (< 10%) and high (≥10%) CVD risk. General linear model and logistic regression model were separately used to assess the associations of lung function with continuous and dichotomous 10-Year CVD Risk.

**Results:**

Cross-sectionally, each 5% decrease in FEV_1_/FVC was associated with a 0.47% increase in 10-Year CVD Risk (*P* < 0.001). The adjusted odds ratio (OR) (95% confidence interval [CI]) for the prevalence of high CVD risk (10-Year CVD Risk≥10%) was 1.12 (1.07, 1.17) corresponding to each 5% decrease in FEV_1_/FVC. The OR (95% CI) for high CVD risk in the lowest group of FEV_1_/FVC (< 70% i.e. chronic obstructive pulmonary disease [COPD]) was 2.37 (1.43, 3.91) when compared with the highest group. Longitudinally, the adjusted risk ratio (RR) (95% CI) for the incidence of high CVD risk was 1.14 (1.03, 1.25) with each 5% decrease in baseline FEV_1_/FVC. Compared with the highest group of FEV_1_/FVC, the RR (95% CI) for high CVD risk in the lowest group (COPD) was 4.06 (1.46, 11.26). Analyses of 10-Year CVD Risk with FVC or FEV_1_ showed similar trends and significant associations (all *P* < 0.05).

**Conclusion:**

Reduced lung function was cross-sectionally and longitudinally associated with increased cardiovascular risk in Chinese general population.

**Electronic supplementary material:**

The online version of this article (10.1186/s12931-018-0920-y) contains supplementary material, which is available to authorized users.

## Background

Cardiovascular diseases (CVD), including coronary disease, cerebrovascular disease, peripheral vascular disease and cardiac failure, are leading causes of morbidity and mortality in China and worldwide [1, 2]. To better prevent and control CVD, a global multivariable risk algorithm based on traditional CVD risk factors including sex, age, total and high density lipoprotein (HDL) cholesterol, systolic blood pressure and treatment for hypertension, smoking and diabetes status, was generated from the Framingham study [3]. The model has been demonstrated to have good discrimination power and be able to estimate overall CVD risk in the next ten years (10-Year CVD Risk) for individuals without CVD [3]. The 10-Year CVD Risk provides useful and elegant composite measures of the classical risk factors for CVD and reflects overall cardiovascular risk of individuals, thus it has been well recognized and widely used in fields of clinic and public health [3–5].

It is well known that cardiovascular and respiratory systems are closely linked with each other in physiology and pathophysiology. Cardiovascular dysfunction could affect lung function, in turn, pulmonary dysfunction may cause adverse cardiovascular outcomes [6, 7]. Pulmonary function, a noninvasive clinical diagnostic parameter, is often used to evaluate the conditions of the respiratory system and identify the severity of pulmonary impairments such as asthma and chronic obstructive pulmonary disease (COPD) [8]. Accumulating evidence suggested that pulmonary dysfunction was positively and independently associated with CVD morbidity [9–15] and mortality [16–20]. A cross-sectional study conducted among 9688 Korean general population without obstructive lung disease found that forced vital capacity (FVC) was inversely related to 10-Year CVD Risk [21]. However, it is still largely unknown whether the reduction of lung function parameters like forced expiratory volume in 1 s (FEV_1_) and the ratio of FEV_1_ to FVC (FEV_1_/FVC) are related to current and future CVD risk such as 10-Year CVD Risk. Further analysis on their associations in both cross-sectional and longitudinal ways will help better clarify and understand the potential effect of lung function decline on CVD.

Therefore, in present study, we investigated and quantified the cross-sectional and longitudinal associations between lung function (including parameters of FEV_1_, FVC and FEV_1_/FVC) and the 10-Year CVD Risk in a Chinese general population.

## Methods

### Study population

The study participants were from the Wuhan-Zhuhai cohort, a Chinese community-based prospective cohort, which has been described previously [22]. Briefly, the cohort was established between 2011 and 2012, comprising 4812 participants aged 18 to 80 years who lived in Wuhan or Zhuhai city in China for more than 5 years. Standardized questionnaires and extensive physical examinations were carried out at baseline and 3 years later. For cross-sectional analysis, participants less than 30 years old (*n* = 260) or previously diagnosed with CVD (*n* = 317) were excluded, as 10-Year CVD Risk estimation was inapplicable for this population [3]. We also excluded 216 subjects with missing data or outliers (>mean ± 3SD) on indexes of blood test, anthropometry, blood pressure or pulmonary function. Finally, a total of 4019 participants were included in our cross-sectional study. For longitudinal analysis, we further excluded 2196 individuals who did not attend physical examinations, or had missing data or outliers on indexes of blood test, anthropometry or blood pressure at 3-year follow-up. After further excluding 752 participants with 10-Year CVD Risk ≥10% at baseline, 1071 subjects were included in our longitudinal study. Individuals included and excluded in our study showed no differences with respect to basic demographic characteristics such as sex, body mass index, smoking status, drinking status, education levels, abdominal obesity, etc. (*P* > 0.05).

### Lung function test

Lung function test was performed in accordance with the recommendation of American Thoracic Society [23]. In brief, spirometry was conducted by specialists using digital spirometers (Chestgraph HI-101, CHEST Ltd., Tokyo, Japan), which were calibrated each day before testing, according to the manufacturer’s instruction. All individuals were suggested not to smoke for at least 1 h and not to have a big meal for 2 h before the test. Each participant was informed to keep a sitting position, wear a nose clip, and then breathe through the mouth-piece after at least 5 min of normal breathing during the testing procedure. Three acceptable volume-time curves of pulmonary function parameters were obtained after three satisfactory blows of each participant performed. Lung function parameters including FVC, FEV_1_ and FEV_1_/FVC were mainly used in our study. COPD was defined as FEV_1_/FVC < 70%, which was further classified into four stages according to Global Initiative for Chronic Obstructive Lung Disease (GOLD) [24]: GOLD 1 (mild: FEV_1_/FVC < 70% and FEV_1_ ≥ 80% predicted), GOLD 2 (moderate: FEV_1_/FVC < 70% and 50% ≤ FEV_1_ < 80% predicted), GOLD 3 (severe: FEV_1_/FVC < 70% and 30% ≤ FEV_1_ < 50% predicted) and GOLD 4 (very severe: FEV_1_/FVC < 70% and FEV_1_ < 30% predicted).

### Ten-year CVD risk calculation and classification

Sex-specific 10-Year CVD Risk was calculated by a multivariable risk factor algorithm that incorporated age, total cholesterol, HDL cholesterol, systolic blood pressure and treatment for hypertension, current smoking, and diabetes status, as described previously [3]. According to the Framingham study [3] and Framingham database derived practice guideline [25], 10-Year CVD Risk was further classified into 2 categories: low (< 10%) and high (≥10%) CVD risk.

### Ascertainment of covariates and CVD risk factors

Body mass index (BMI) was calculated by dividing weight (kg) by the squared value of height (m). Active physical activity was defined as regular exercise ≥2 times per week and each time ≥ 20 min within the last 6 months. Education degree was classified into 3 levels: middle school or below, high school, and university or above. Smokers comprised both current and former smokers, and smoking amount (pack-years) for each smoker was computed as packs of cigarettes per day multiplied by years of smoking. Participants were divided into drinkers (including current and former drinkers) and nondrinkers. Abdominal obesity was defined as waist circumference ≥ 90 cm for men or ≥ 80 cm for women. Blood lipids and fasting glucose levels were determined in the clinical laboratory of hospitals. Blood pressure was measured on the right arm of the seated participant with a validated automatic oscillometric device. Diabetes was defined as fasting plasma glucose ≥7.0 mmol/L, or taking oral hypoglycemic medication or insulin, or self-reported physician-diagnosed diabetes.

### Statistical analyses

Subjects were divided into four groups based on quartiles (Q) of FVC or FEV_1_ or clinical thresholds of FEV_1_/FVC level (L1 < 70%; L2 70% ~ < 80%; L3 80% ~ < 90%; L4 ≥ 90%), for which 70% is a clinical threshold of COPD diagnosis and 80% is a critical value of normal lung function. Baseline characteristics across groups of FEV_1_/FVC were compared by variance analysis for continuous variables and Cochran-Armitage trend test for dichotomous variables. Analysis of covariance was used to compare 10-Year CVD Risk by groups of lung function parameters, with adjusting for gender, height, weight, abdominal obesity, smoking amount, drinking status, low density lipoprotein (LDL), physical activity, city and education levels. Age was not included in the statistic models for adjustment again, because as a dependent variable, 10-Year CVD Risk was estimated by a multivariable risk factor algorithm where age has been included, further adjustment for age in the statistic models again will lead to overcorrection and conservative association between lung function and CVD risk.

Association of lung function with continuous 10-Year CVD Risk was assessed using general linear model, with adjustment for potential confounders as mentioned above. The association was quantified by using estimated changes and 95% confidence intervals (CIs) of 10-Year CVD Risk with each 5% decrease of FEV_1_/FVC or each 50-mL decrease of FVC or FEV_1_ in continuous analyses. We also estimated changes (95% CI) of 10-Year CVD Risk across groups of lung function parameters in categorical analyses with the highest group (L4 or Q4) as the reference.

Logistic regression model was used to calculate the odds ratios (ORs), risk ratios (RRs) and 95% CI for dichotomous 10-Year CVD Risk (individuals with 10-Year CVD Risk ≥10% were regarded as cases) according to the decreasing of baseline lung function level, with adjusting for potential covariates as mentioned above. All statistical analyses were performed with SAS version 9.4 (SAS Institute, Cary, NC), and all *p*-values were two sided with a significant level at 0.05.

## Results

### Baseline characteristics

The baseline characteristics of the participants based on groups of FEV_1_/FVC are presented in Table 1. The mean age of 4019 participants (1304 men; 32.45%) was 53.98 years. Without adjustment for any confounder, the number of smokers, drinkers and participants with high 10-Year CVD Risk (≥10%), as well as age, smoking amount, low density lipoprotein and 10-Year CVD Risk significantly increased across decreasing FEV_1_/FVC groups (*P* < 0.001). In further analysis with COPD patients (group L1: FEV_1_/FVC < 70%), we found an upward trend of 10-Year CVD Risk as the progresses of COPD (from GOLD 1 to GOLD 4, *P*_trend_ = 0.041) (Table S1). And COPD patients with older age, male sex, smoking or drinking habits achieved higher CVD risk (*P* < 0.05) (Additional file 1: Table S1).

**Table 1:** Baseline characteristics of study participants by groups of FEV_1_/FVC and in all participants (*N* = 4019)

Variables		FEV_1_/FVC (%)				
	All participants	L4 (≥90)	L3 (80 ~ < 90)	L2 (70 ~ < 80)	L1 (< 70)	*P*_trend_
No. subjects	4019	1606	1664	648	101	
No. subjects in Wuhan city	2536 (63.10)	855 (53.24)	1085 (65.2)	511 (78.86)	85 (84.16)	< 0.001
Age, years	53.98 ± 11.21	52.14 ± 11.02	54.23 ± 11.02	56.69 ± 11.16	61.51 ± 10.58	< 0.001
male sex	1304 (32.45)	485 (30.20)	509 (30.59)	261 (40.28)	49 (48.51)	< 0.001
Body mass index, kg/m^2^	24.05 ± 3.36	24.08 ± 3.45	24.12 ± 3.31	23.93 ± 3.27	23.30 ± 3.23	0.105
Education levels						
Middle school or below	2505 (62.33)	958 (59.65)	1042 (62.62)	428 (66.05)	77 (76.24)	< 0.001
High school	1100 (27.37)	457 (28.46)	458 (27.52)	167 (25.77)	18 (17.82)	0.035
University or above	414 (10.30)	191 (11.89)	164 (9.86)	53 (8.18)	6 (5.94)	0.002
Physical activity	1967 (48.94)	791 (49.25)	836 (50.24)	298 (45.99)	42 (41.58)	0.120
Smokers^a^	886 (22.05)	307 (19.12)	351 (21.09)	192 (29.63)	36 (35.64)	< 0.001
Smoking amount, pack-years^b^	5.29 ± 14.09	4.26 ± 12.79	4.71 ± 13.12	8.38 ± 17.37	11.45 ± 20.29	< 0.001
Drinkers^a^	714 (17.77)	257 (16.00)	276 (16.59)	154 (23.77)	27 (26.73)	< 0.001
Abdominal obesity	1795 (44.66)	707 (44.02)	774 (46.51)	280 (43.21)	34 (33.66)	0.392
LDL, mmol/L	3.10 ± 1.02	2.98 ± 1.02	3.15 ± 1.02	3.23 ± 1.02	3.19 ± 0.89	< 0.001
10-Year CVD Risk, %	10.24 ± 9.11	9.16 ± 8.50	10.21 ± 9.10	12.25 ± 9.81	15.11 ± 10.25	< 0.001
10-Year CVD Risk						
Low (< 10%)	2452 (61.01)	1050 (65.38) ()(65.38)	1033 (62.08)	329 (50.77)	40 (39.60)	< 0.001
High (≥10%)	1567 (38.99)	556 (34.62)	631 (37.92)	319 (49.23)	61 (60.40)	< 0.001

Abbreviations: *FEV*_*1*_*/FVC* the ratio of forced expiratory volume in the 1 s to forced vital capacity, *LDL* low density lipoprotein

Values are n (%) or mean ± SD

^a^Smokers/drinkers included both current and former smokers/drinkers

^b^Smoking amount was calculated among both current and former smokers

### Association of pulmonary function with 10-year CVD risk

**Fig. 1:**
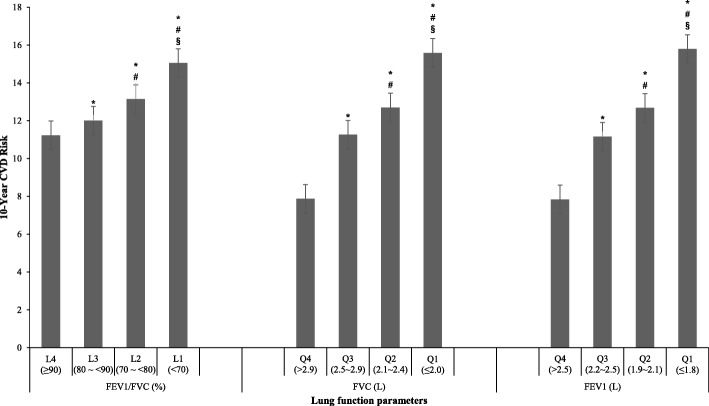
The 10-Year CVD Risk in all participants by groups of lung function parameters using analysis of covariance. Abbreviations: FEV_1_, forced expiratory volume in 1 s; FVC, forced vital capacity; FEV_1_/FVC, the ratio of FEV_1_ to FVC. ^*^ Significant differences between L4/Q4 and any other lung function group at *P* < 0.05; ^**#**^ significant differences between L3/Q3 and any other lung function group at *P* < 0.05; ^**§**^ significant differences between L2/Q2 and any other lung function group at *P* < 0.05. Adjusted for gender (male/female), height (continuous, m), weight (continuous, kg), abdominal obesity (yes/no), smoking amount (continuous, pack-year), drinking status (drinker/nondrinker), low density lipoprotein (continuous, mmol/L), physical activity (active/inactive), city (Wuhan/Zhuhai) and education levels (middle school or below, high school, university or above)

With adjustment for potential covariates, leastsquares means of 10-Year CVD Risk by groups of lung function parameters at baseline are shown in Fig. 1. The highest 10-Year CVD Risk was observed in the lowest group of lung function parameters (L1 [COPD group] or Q1) when compared with those in other groups. Upward trend of 10-Year CVD Risk was significantly associated with decreased lung function groups.

Table 2 presents an inverse relationship between lung function and continuous 10-Year CVD Risk at baseline. After adjusting for potential confounders, each 5% decrease in FEV_1_/FVC was associated with a 0.47% increase in 10-Year CVD Risk (*P* < 0.001). Remarkably monotonic increase of 10-Year CVD Risk was shown when FEV_1_/FVC gradually decreased (*P*_trend_ < 0.001). Similar trends and significant associations were also observed between FVC and FEV_1_ and 10-Year CVD Risk (Table 2).

**Table 2:** Association between lung function and 10-Year CVD Risk (*N* = 4019)

Lung function parameters	Estimated changes (95% CI) by continuous lung function parameters^a^	Estimated changes (95% CI) by groups of lung function parameters				*p*_trend_
FEV_1_/FVC (%)		L4 (≥90)	L3 (80 ~ < 90)	L2 (70 ~ < 80)	L1 (< 70)	
	0.47 (0.34, 0.60)	0.00 (ref.)	0.78 (0.28, 1.28)	1.91 (1.24, 2.59)	3.82 (2.34, 5.30)	< 0.001
FVC (L)		Q4 (> 2.9)	Q3 (2.5~ 2.9)	Q2 (2.1~ 2.4)	Q1 (≤2.0)	
	0.24 (0.22, 0.26)	0.00 (ref.)	3.40 (2.71, 4.08)	4.83 (4.08, 5.58)	7.72 (6.92, 8.51)	< 0.001
FEV_1_ (L)		Q4 (> 2.5)	Q3 (2.2~ 2.5)	Q2 (1.9~ 2.1)	Q1 (≤1.8)	
	0.28 (0.25, 0.30)	0.00 (ref.)	3.33 (2.65, 4.01)	4.85 (4.10, 5.60)	7.96 (7.21, 8.71)	< 0.001

Abbreviations: *FEV*_*1*_ forced expiratory volume in 1 s; *FVC* forced vital capacity, *FEV*_*1*_*/FVC* the ratio of FEV_1_ to FVC, *CI* confidence interval

Adjusted for gender (male/female), height (continuous, m), weight (continuous, kg), abdominal obesity (yes/no), smoking amount (continuous, pack-year), drinking status (drinker/nondrinker), low density lipoprotein (continuous, mmol/L), physical activity (active/inactive), city (Wuhan/Zhuhai) and education levels (middle school or below, high school, university or above)

^a^Estimated changes of 10-Year CVD Risk were calculated by each 5% decrease of FEV_1_/FVC or each 50-mL decrease of FVC or FEV_1_ in continuous analyses

Table 3 shows a negative association between lung function and dichotomous10-Year CVD Risk at baseline. After adjusting for potential confounders, the OR (95%CI) for the prevalence of high CVD risk (10-Year CVD Risk ≥10%) was 1.12 (1.07, 1.17) with each 5% decrease in FEV_1_/FVC. Compared with the highest FEV_1_/FVC group, multi-variate adjusted ORs (95%CI) gradually increased when lung function decreased. From the second highest to the lowest FEV_1_/FVC group (COPD), they were 1.11 (0.94, 1.31), 1.63 (1.31, 2.03), 2.37 (1.43, 3.91) for high CVD risk. Similar trends and significant relationships were also shown between FVC and FEV_1_ and 10-Year CVD Risk (Table 3).

**Table 3:** Odds ratios for 10-Year CVD Risk according to lung function parameters (*N* = 4019)

Variables	ORs (95% CI) by Continuous lung function^a^	ORs (95% CI) by groups of lung function parameters				*p*_*trend*_
FEV_1_/FVC (%)		L4 (≥90)	L3 (80 ~ < 90)	L2 (70 ~ < 80)	L1 (< 70)	
N (≥10% / < 10%)	1567/2452	556/1050	631/1033	319/329	61/40	
Adjusted OR (95% CI)	1.12 (1.07, 1.17)	1.00 (ref.)	1.11 (0.94, 1.31)	1.63 (1.31, 2.03)	2.37 (1.43, 3.91)	< 0.001
FVC (L)		Q4 (> 2.9)	Q3 (2.5~ 2.9)	Q2 (2.1~ 2.4)	Q1 (≤2.0)	
N (≥10% / < 10%)	1567/2452	358/526	347/683	343/715	519/528	
Adjusted OR (95% CI)	1.07 (1.06, 1.08)	1.00 (ref.)	2.34 (1.79, 3.06)	4.33 (3.18, 5.90)	9.82 (7.11, 13.56)	< 0.001
FEV_1_ (L)		Q4 (> 2.5)	Q3 (2.2~ 2.5)	Q2 (1.9~ 2.1)	Q1 (≤1.8)	
N (≥10% / < 10%)	1567/2452	363/554	311/690	284/608	609/600	
Adjusted OR (95% CI)	1.09 (1.08, 1.10)	1.00 (ref.)	2.33 (1.77, 3.07)	4.10 (3.00, 5.60)	10.37 (7.58, 14.19)	< 0.001

Abbreviations: *FEV*_*1*_ forced expiratory volume in 1 s, *FVC* forced vital capacity, *FEV*_*1*_*/FVC* the ratio of FEV_1_ to FVC *OR* odds ratio; *CI* confidence interval

Adjusted for gender (male/female), height (continuous, m), weight (continuous, kg), abdominal obesity (yes/no), smoking amount (continuous, pack-year), drinking status (drinker/nondrinker), low density lipoprotein (continuous, mmol/L), physical activity (active/inactive), city (Wuhan/Zhuhai) and education levels (middle school or below, high school, university or above)

^a^Odds ratios were estimated by each 5% decrease of FEV_1_/FVC or each 50-mL decrease of FVC or FEV_1_ in continuous analyses

After 3 years of follow-up, we recalculated the 10-Year CVD Risk for the 1071 participants included in our longitudinal study, and 214 incident cases of high CVD risk (10-Year CVD Risk ≥10%) were identified. RRs for the incidence of high CVD risk are shown in Table 4. A negative association between baseline lung function and incidence of high CVD risk was observed. The RR (95%CI) was 1.14 (1.03, 1.25) corresponding to each 5% decline in FEV1/FVC. The categorical analysis also showed a significant monotonic RR increase of high CVD risk as the decreasing of FEV_1_/FVC groups (*P*_trend_ = 0.021). Compared with the highest FEV_1_/FVC group, the RRs (95%CI) for high CVD risk were 1.15 (0.80, 1.65), 1.41 (0.85, 2.34), 4.06 (1.46, 11.26) from the second highest to the lowest FEV_1_/FVC group (COPD). Similarly, significant negative associations of FVC and FEV_1_ with 10-Year CVD Risk were observed with all *P* and *P*_trend_ < 0.001 (Table 4).

**Table 4:** Risk ratios for 10-Year CVD Risk according to lung function parameters (*N* = 1071)

Variables	RRs (95% CI) by Continuous lung function^a^	RRs (95% CI) by groups of lung function parameters				*p*_*trend*_
FEV_1_/FVC (%)		L4 (≥90)	L3 (80 ~ < 90)	L2 (70 ~ < 80)	L1 (< 70)	
N (≥10% / < 10%)	214/857	82/377	90/361	33/110	9/9	
Adjusted RR (95% CI)	1.14 (1.03, 1.25)	1.00 (ref.)	1.15 (0.80, 1.65)	1.41 (0.85, 2.34)	4.06 (1.46, 11.26)	0.021
FVC (L)		Q4 (> 2.8)	Q3 (2.5~ 2.8)	Q2 (2.1~ 2.4)	Q1 (≤2.0)	
N (≥10% / < 10%)	214/857	60/197	29/211	61/266	64/183	
Adjusted RR (95% CI)	1.04 (1.02, 1.06)	1.00 (ref.)	1.10 (0.58, 2.10)	2.07 (1.11, 3.85)	3.19 (1.68, 6.06)	< 0.001
FEV_1_ (L)		Q4 (> 2.5)	Q3 (2.2~ 2.5)	Q2 (1.9~ 2.1)	Q1 (≤1.8)	
N (≥10% / < 10%)	214/857	46/180	43/235	49/243	76/199	
Adjusted RR (95% CI)	1.07 (1.05, 1.09)	1.00 (ref.)	2.46 (1.28, 4.76)	3.33 (1.63, 6.81)	6.49 (3.19, 13.18)	< 0.001

Abbreviations: FEV_1_, forced expiratory volume in 1 s; FVC, forced vital capacity; FEV_1_/FVC, the ratio of FEV_1_ to FVC; RR, risk ratio; CI, confidence interval

Adjusted for gender (male/female), height (continuous, m), weight (continuous, kg), abdominal obesity (yes/no), smoking amount (continuous, pack-year), drinking status (drinker/nondrinker), low density lipoprotein (continuous, mmol/L), physical activity (active/inactive), city (Wuhan/Zhuhai) and education levels (middle school or below, high school, university or above)

^a^Risk ratios were estimated by each 5% decrease of FEV_1_/FVC or each 50-mL decrease of FVC or FEV_1_ in continuous analyses

## Discussion

In the present study, negative cross-sectional and longitudinal associations were identified between lung function and 10-Year CVD Risk. After adjusting for potential confounders, increased prevalence and incidence of high CVD risk (10-Year CVD Risk ≥10%) were observed with the decline of lung function level. Additionally, when 10-Year CVD Risk was further classified into ≥6%/< 6% or > 20%/≤ 20% as also proposed by the Framingham study [3] and practice guideline derived from Framingham database [25], similar trends and significant associations with lung function were also found (data not shown). Our findings help to understand the correlation between lung function and current and future CVD risk. They also have significant implications for public health. Lung function test is a noninvasive clinical diagnostic method and is easy taken after routing training. The significant relationship between reduced lung function and increased CVD risk in ten years indicated that improving lung function or preventing lung function decline may help to prevent CVD.

Lung function has been linked to CVD risk in previous studies [21, 26, 27]. The 4th Korea National Health and Nutrition Examination Survey found that FVC decline was cross-sectionally associated with increased 10-Year CVD Risk [21]. Similarly, a study by Arcari et al. on Italian general population showed that FVC or FEV_1_ reduction was cross-sectionally associated with elevated 10-Year CVD Risk [27]. However, they did not find the association between FEV_1_/FVC, a mainly clinical diagnostic indicator for obstructive lung diseases such as COPD, and 10-Year CVD Risk, which is inconsistent with our findings. In our study, we found that not only FVC and FEV_1_ but also FEV_1_/FVC reduction was cross-sectionally and longitudinally associated with increased10-Year CVD Risk. Such discrepancy between our study and the published data may be partly due to the differences in race, genetics and lifestyles of the study population. For example, as a common risk factor for CVD, cigarette smoking rate was 22.05% in our study, lower than the mean smoking rate (28.10%) for adults in China [28]. Physical activity rate, a protective factor of CVD, was 48.94% in our study, much higher than the average level (11.90%) of Chinese adults [29]. These factors may lead to more obvious effect of lung function decline on CVD. Additionally, compared with the participants in our study (average BMI: 24.05 kg/m^2^), the subjects included in Arcari’s study had higher BMI with a mean value of 27.6 kg/m^2^, which was considered as overweight and on the brink of obesity [30]. As risk factors for CVD [25, 31], overweight and obesity may partly conceal the effects of FEV_1_/FVC on the risk of CVD in Arcari’s study.

Furtherly, we noted that participants with COPD (FEV_1_/FVC < 70%) achieved the highest 10-Year CVD Risk in our present study. This result is consistent with those reported by Ford and colleagues. They found that aged adults with obstructive or restrictive impairment had an increased 10-Year CVD Risk compared with those with normal lung function [26]. Besides, accumulating evidence suggested that COPD patients have an elevated risk of CVD and cardiovascular death, and nearly two fifths of COPD patients die of CVD [17, 18, 32].

The underlying mechanisms between pulmonary dysfunction and CVD remain incompletely understood. Generally, pulmonary and cardiovascular functions are closely related in both physiological and pathological conditions. At the circumstance of lung function decline, cardiac pumping function has to increase compensatorily to ensure the body’s oxygen need, which may result in cardiac and vascular overloads, and even cardiovascular injuries [33, 34]. If these situations were not improved timely, cardiovascular events might occur in the near future [33]. Additionally, shared risk factors may partly explain the association between poor lung function and elevated CVD risk [7]. It is well documented that several risk factors for lung function reduction, such as aging and smoking, are also well-established risk factors for CVD [3]. And traditional cardiovascular risk factors such as hypertension, dyslipidemia and diabetes mellitus are common in subjects with lung function impairment including COPD [35]. Air pollutants like particulate matters and polycyclic aromatic hydrocarbons are notable risk factors for both poor lung function and cardiovascular events [36–38]. However, the still observed association between lung function and CVD after adjusting for shared risk factors suggested the involvement of additional explanations [20].

Further explanations could be inflammation and oxidative stress, which were reported to have important contributions to both lung function decline and CVD risk increase [7]. Evidence has shown that inflammation markers such as C-reactive protein, fibrinogen and inflammation-sensitive plasma proteins were involved in the inverse relationship between lung function and CVD risk [20, 21, 39]. As a major driving mechanism in the pathophysiology of lung function impairment, elevated oxidative stress in local pulmonary microenvironment may directly affect cardiovascular system [7]. Previous studies suggested that oxidative stress may cause vascular dysfunction through inactivating the endothelial-derived nitric oxide by superoxide anion [40]. Generation of reactive oxygen species (ROS) could promote inflammation in the vascular wall by inducing the production of pro-inflammatory genes and cytokines via the activation of NF-κB [41], whereas in turn, inflammatory cytokines (TNF-α, IL-6, etc.) could increase ROS production by NADPH oxidases [42, 43], causing a vicious circle that exacerbates vascular dysfunction [44]. Moreover, oxidative stress and inflammation could also alter the vascular structure by promoting vascular remodeling, stiffness and atherosclerosis [44–46]. Therefore, the inflammation and increased oxidative stress in pulmonary dysfunction may independently increase CVD risk by altering vascular structure and promoting vascular dysfunction and insufficiency [7]. Besides, evidence has shown that pulmonary dysfunction specific inflammation and oxidative stress may elevate cardiovascular risk also through increasing susceptibility to thrombotic or embolic events [47–49].

The strengths of our study include a relative large study population and a 3-year follow-up. Based on that, we could investigate the cross-sectional relationship between lung function and CVD risk and longitudinally evaluate the changes after 3 years. And to our knowledge, it is the first prospective study to investigate the relationship between lung function and 10-Year CVD Risk. However, there are still several limitations. First, rather than clinically diagnosed CVD, we evaluated the risk of CVD using a global multivariable risk algorithm, which was clinically used to estimate the 10-Year CVD Risk of individuals. Nevertheless, as an available endpoint, 10-Year CVD Risk do provide useful and elegant composite measures of the classical risk factors for CVD and represent individuals’ overall cardiovascular risk. Moreover, we are not unique in using 10-Year CVD Risk as a composite measure of CVD risk (endpoint), and a similar method was also taken by studies draw from Cardiovascular Risk Factor Multiple Evaluation in Latin America Study [50], Louisville Healthy Heart Study [51], Lifestyle Interventions and Independence for Elders Study [52], International Mobility in Aging Study [53], etc. Second, the CVD risk was merely estimated at baseline and at 3 years of follow-up, and the follow-up time may be relatively short. Further long-time longitudinal study may help to accurately evaluate such relationship.

## Conclusions

Our study clearly demonstrated that reduced lung function was cross-sectionally and longitudinally associated with increased CVD risk in a general Chinese population. It suggests that improve lung function or prevent lung function decline may help to prevent CVD. Further studies with long-time follow-up are needed to validate our findings and illuminate the potential mechanisms.

## Additional file

Additional file 1: **Table S1.** Ten-Year CVD Risk by GOLD classification and selected baseline characteristics in COPD patients (*N* = 101). (DOCX 17 kb)

## Abbreviations

BMI: body mass index
CI: confidence interval
COPD: chronic obstructive pulmonary disease
CVD: cardiovascular diseases
FEV_1_: forced expiratory volume in 1 s
FEV_1_/ FVC: the ratio of FEV_1_ to FVC
FVC: forced vital capacity
GOLD: global initiative for chronic obstructive lung disease
HDL: high density lipoprotein
IL-6: Interleukin-6
LDL: low density lipoprotein
NADPH: Nicotinamide adenine dinucleotide phosphate
NF-κB: nuclear factor kappa B
OR: odds ratio
ROS: reactive oxygen species
RR: risk ratio
TNF-α: tumour necrosis factor alpha
