# Supplementary material for: Association of lung function with cardiovascular risk: a cohort study
Source: Respir Res. 2018 Nov 6;19:214. doi: 10.1186/s12931-018-0920-y (PMC6219159; doi:10.1186/s12931-018-0920-y)
Supplement: Supplementary file 1 — Table S1. Ten-Year CVD Risk by GOLD classification and selected baseline characteristics in COPD patients (N = 101). (DOCX 17 kb) [file 12931_2018_920_MOESM1_ESM.docx]

**Table S1. Ten-Year CVD Risk by GOLD classification and selected baseline characteristics in COPD patients (N=101).**

| **Variables** | **10-Year CVD Risk** | ***P*** |
| --- | --- | --- |
| All COPD patients (N=101) | 15.11±10.25 |  |
| GOLD 1 (n=21) | 10.14±8.82 | **0.041** |
| GOLD 2 (n=49) | 16.70±10.36 |  |
| GOLD 3 (n=24) | 15.43±9.56 |  |
| GOLD 4 (n=7) | 17.83±12.9 |  |
| Age, years |  | **<0.001** |
| <65 (n=62) | 10.54±7.81 |  |
| ≥65 (n=39) | 22.39±9.49 |  |
| Gender |  | **<0.001** |
| Male (n=49) | 20.68±9.34 |  |
| Female (n=52) | 9.87±8.13 |  |
| Body mass index, kg/m^2^ |  | 0.337 |
| <24 (n=59) | 14.28±10.15 |  |
| ≥24 (n=42) | 16.28±10.40 |  |
| Smoker^*^ |  | **<0.001** |
| Yes (n=36) | 21.52±8.87 |  |
| No (n=65) | 11.56±9.23 |  |
| Drinker^*^ |  | **0.022** |
| Yes (n=27) | 18.96±8.71 |  |
| No (n=74) | 13.71±10.46 |  |
| Abdominal obesity |  | 0.317 |
| Yes (N=34) | 16.55±9.21 |  |
| No (N=67) | 14.38±10.73 |  |

Abbreviations: COPD, chronic obstructive pulmonary disease; GOLD, global initiative for chronic obstructive lung disease.

^*^Smokers/drinkers included both current and former smokers/drinkers.
